# The Superior Fronto-Occipital Fasciculus in the Human Brain Revealed by Diffusion Spectrum Imaging Tractography: An Anatomical Reality or a Methodological Artifact?

**DOI:** 10.3389/fnana.2017.00119

**Published:** 2017-12-13

**Authors:** Yue Bao, Yong Wang, Wei Wang, Yibao Wang

**Affiliations:** Department of Neurosurgery, The First Affiliated Hospital of China Medical University, Shenyang, China

**Keywords:** superior fronto-occipital fasciculus, diffusion spectrum imaging, tractography, white matter, fiber dissection

## Abstract

The existence of the superior fronto-occipital fasciculus (SFOF) in the human brain remains controversial. The aim of the present study was to clarify the existence, course, and terminations of the SFOF. High angular diffusion spectrum imaging (DSI) analysis was performed on six healthy adults and on a template of 842 subjects from the Human Connectome Project. To verify tractography results, we performed fiber microdissections of four post-mortem human brains. Based on DSI tractography, we reconstructed the SFOF in the subjects and the template from the Human Connectome Project that originated from the rostral and medial parts of the superior and middle frontal gyri. By tractography, we found that the fibers formed a compact fascicle at the level of the anterior horn of the lateral ventricle coursing above the head of caudate nucleus, medial to the corona radiate and under the corpus callosum (CC), and terminated at the parietal region via the lower part of the caudate nucleus. We consider that this fiber bundle observed by tractography is the SFOF, although it terminates mainly at the parietal region, rather than occipital lobe. By contrast, we were unable to identify a fiber bundle corresponding to the SFOF in our fiber dissection study. Although we did not provide definite evidence of the SFOF in the human brain, these findings may be useful for future studies in this field.

## Introduction

The superior fronto-occipital fasciculus (SFOF) was first described as a longitudinal ridge in autopsies of acallosal patients by Eichler (1878). Since then, numerous post-mortem studies have proposed a range of descriptions, hypotheses and terminology of the SFOF. Sachs (1892) made the important observation that the SFOF of acallosal patients was composed of callosal fibers that failed to pass through the interhemispheric midline, which was verified in later more advanced studies (Forkel et al., 2014). As well as confirming that the SFOF represented an association bundle connecting frontal and occipital regions in the acallosal brain, Onufrowicz (1887) and Dejerine (1895) extended these findings to the healthy brain. Thus, the prevailing consensus proposed by Dejerine is that the SFOF is a distinct 2–3 mm wide white matter located medial to the corona radiate, superolateral to the caudate nucleus, and ventrolateral to the corpus callosum (CC), and which interconnects the frontal and occipital lobes as association fibers.

Using isotope tract-tracing in the monkey brain, Schmahmann and Pandya confirmed that the SFOF courses above the caudate nucleus, medial to the corona radiate, and lateral and ventral to the CC, connects the parietal lobe with the frontal lobe, and plays a role in the rapid top-down modulation of visual processing and spatial aspects of cognitive processing (Bar et al., 2006; Schmahmann and Pandya, 2007). Axonal tracing results of non-human primates are generally considered the “gold standard” guide for human white matter connective patterns (Thiebaut de Schotten et al., 2012). However, with evolution, the frontal and parieto-occipital regions of the human brain differ markedly from their monkey counterparts. Thus, extrapolating results from monkey studies to humans can be misleading (Rilling et al., 2008). Indeed, despite being well described in human, the inferior fronto-occipital fasciculus (IFOF) does not exist in the monkey brain (Schmahmann et al., 2007; Forkel et al., 2014).

In the last decade, the development of diffusion tensor imaging (DTI) and diffusion tractography have provided a unique opportunity to study white matter architecture *in vivo* (Dick and Tremblay, 2012). Indeed, a number of fiber tractography DTI studies have identified the SFOF in the human brain (Catani et al., 2002; Wakana et al., 2004; Makris et al., 2007). By contrast, using tractography with DTI Meola et al. (2015) did not identify any tracts in the previously described SFOF location. Further, the SFOF was not identified by tractography with a diffusion imaging approach based on spherical deconvolution (Forkel et al., 2014), and the “SFOF” was proposed to represent a branch of the superior longitudinal fasciculus (SLF). Recent microsurgical anatomical studies in post-mortem human brains also reported that the SFOF is actually the superior thalamic peduncle (STP), rather than association fibers (Türe et al., 1997; Meola et al., 2015).

These contrasting fiber dissection and *in vivo* tractography findings may be attributed to the methodological limitations of these techniques. For example, DTI-based tractography is unable to resolve multiple fiber orientations within an magnetic resonance imaging (MRI) voxel, and as such, cannot resolve fiber crossings either as tract intersections in the white matter or the intricate architecture of the gray matter (Mori and van Zijl, 2002), resulting in artifacts and false tracts (Le Bihan et al., 2006; Fernandez-Miranda, 2013; Wang et al., 2013). In recent years, high-angular-resolution diffusion imaging (HARDI) has been used to overcome the deficiencies of DTI. HARDI is based on diffusion spectrum imaging (DSI), and is reconstructed by generalized Q-sampling imaging (Yeh et al., 2010). Thus, in the present study we mapped the SFOF in six healthy subjects, as well as using a template approach (HCP-842 Atlas), using HARDI tractography. We also performed a fiber dissection study with cross-validation to tractography findings. Our findings provide further clarification of the anatomy of the SFOF, and may be beneficial for planning approaches for neurosurgical procedures.

## Materials and Methods

### Participants

Six neurologically healthy adults (three men; all right handed; age range: 26–43 years) were included in this study. All procedures were approved by the Ethics Committee of the First Affiliated Hospital of China Medical University, and written consent was obtained from all participants before testing. All subjects gave written informed consent in accordance with the Declaration of Helsinki. The protocol was approved by the the Ethics Committee of the First Affiliated Hospital of China Medical University.

We also performed fiber tracking on a template of 842 subjects from the Human Connectome Project (HCP-842), which represents the largest and highest quality data set available to date. The Wu-Minn HCP consortium is an ongoing project led by Washington University, University of Minnesota and Oxford University, involving a systematic effort to map macroscopic human brain circuits and their relationship to behavior in a large population of healthy adults. Data from a total of 900 subjects were released for the first four quarters (Q1–Q4, 2015), and 842 subjects had diffusion scans. The reconstructed data of the 842 subjects were averaged to create a representative template (the HCP 842-subject template is freely downloadable at: http://dsi-studio.labsolver.org/download-images).

### Image Acquisition and Reconstruction

For the subject-specific approach, DSI data were acquired on 3T Tim Trio System (Siemens) using a 32-channel coil. This involved a 43-min, 257-direction scan using a twice-refocused spin-echo EPI sequence and multiple *q* values (Wedeen et al., 2005; repetition time [TR] = 9.916 ms, echo time [TE] = 157 ms, voxel size = 2.4 × 2.4 × 2.4 mm, field of view = 231 × 231 mm, *b*_max_ = 7000 s/mm^−2^). We also included the high-resolution anatomical imaging as anatomical comparisons, employing a 9-min T_1_-weighted axial magnetization prepared rapid gradient echo sequence (TE = 2.63 ms, TR = 2110 ms, 176 slices, flip angle = 8°, field of view = 256 × 256 mm^−2^, voxel size = 0.5 × 0.5 × 1.0 mm^3^). DSI data were reconstructed using a generalized Q-sampling imaging approach (Yeh et al., 2010). The orientation distribution functions were reconstructed to 362 discrete sampling directions and a mean diffusion distance of 1.2 mm.

### The Human Connectome Project 842-Subject Template

The HCP-842 Atlas was constructed using diffusion MRI data from a total of 842 subjects from the HCP (2015 Q4, 900-subject release). The 842 subjects underwent diffusion scans in a Siemens 3T Skyra scanner using a two dimensional spin-echo single-shot multiband EPI sequence with a multi-band factor of three and a monopolar gradient pulse (Sotiropoulos et al., 2013). The spatial resolution was 1.25 mm isotropic (TR = 5500 ms, TE = 89 ms). A multishell diffusion scheme was used for acquiring the diffusion images, with *b*-values of 1000, 2000 and 3000 s/mm^2^. The total number of diffusion sampling directions was 270. The total scanning time was approximately 55 min (Van Essen et al., 2013). Diffusion data were collected using a Q-sampling imaging approach.

### Fiber Tracking and Analysis

DSI Studio (open-source diffusion MRI analysis tool, freely downloadable at http://dsi-studio.labsolver.org) was used for fiber tracking. A whole brain seeding approach using multiple region of interest (ROI) masks was performed. In voxels with multiple fiber orientations, fiber tracking was initiated separately for each orientation, and fiber progression continued with a step size of 1.2 mm, minimum fiber length of 20 mm and turning angle threshold of 60°. If multiple fiber orientations existed in the current progression location, the fiber orientation that was nearest to the incoming direction and formed a turning angle smaller than 60° was selected to determine the next moving direction (Fernandez-Miranda et al., 2015). To smooth each track, the next moving directional estimate of each voxel was weighted by 20% of the previous incoming direction and 80% of the nearest fiber orientation. This progression was repeated until the quantitative anisotropy of the fiber orientation dropped below a preset threshold (0.03–0.06 depending on the subject) or there was no fiber selected within the 60° angular range in the progression (Yeh et al., 2013). Once tracked, all streamlines were saved in the TrackVis file format. For comparison, FreeSurfer^1^ was used to segment cortical gyral ROIs based on previous brain atlases using each participant’s T_1_-weighted magnetization prepared rapid axial gradient echo image (Desikan et al., 2006).

**Figure 1:**
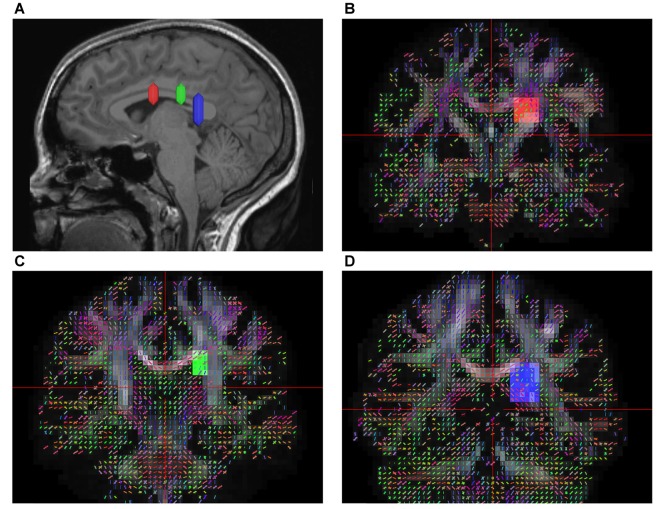
*In vivo* fiber tractography of the superior fronto-occipital fasciculus (SFOF). **(A)** Sagittal plane; lateral view of the hemisphere showing the rostrocaudal level of the three region of interests (ROIs), the three ROIs were located at the level of the anterior commissure, the thalamus and the pineal. **(B–D)** Coronal view on the QA map; all three ROIs were located under the corpus callosum (CC), lateral to the lateral ventricle and medial to the internal capusule.

According to prior anatomical studies reported by Dejerine (1895) and experimental animal studies (Schmahmann and Pandya, 2007), the SFOF is a distinct fiber bundle coursing between the ependyma of the lateral ventricle and the internal capsule, superolateral to the caudate nucleus, medial to the corona radiate, and under the CC. Using this information, we tried to reconstruct the SFOF. Three ROIs were drawn on the coronal quantitative anisotropy color map to select the anteroposterior direction fibers that pass through all three ROIs. On the sagittal plane, the rostral ROI was located at the level of the anterior commissure, the middle ROI was located at the level of the thalamus, and the caudal ROI was located at the level of the pineal. On the coronal plane, all three ROIs were located superolateral to the caudate nucleus, medial to the internal capsule and under the CC (Figure 1).

We also reconstructed the SLF-II, IFOF and the cingulum bundle (CB) to analyze the spatial relationship of the SFOF with these association fibers. For the SLF, the angular gyrus, supramarginal gyrus, superior parietal lobule, and precuneus were used as seed regions, and an ROI mask was drawn along the precentral region on the coronal plane (Wang et al., 2016). For the IFOF, the frontal lobe was selected as the seeding region, and two ROIs were drawn at the level of the central sulcus and ventral part of the external capsule on the coronal plane (Wu et al., 2016a). For the CB, the cingulate cortex was selected as the seeding region (Wu et al., 2016b).

### Fiber Dissection Technique

Eight hemispheres from four normal brains (36–70 years old, three men) were obtained at routine autopsy. Operations were approved by the ethics committee at China Medical University. The fiber dissection study was performed using the Klingler technique (Agrawal et al., 2011). The specimens were fixed in a 10% formalin aqueous solution for at least 4 weeks, and we then removed the vessels, arachnoid and pia-matter, which were frozen for an additional 2 weeks at −16°C. Because of the freezing progress, water crystallization disrupted the structure of the gray matter, allowing us to peel off the cortex from brain surface and isolate the fiber bundles in their glial sheets. We performed fiber dissection studies at the Surgical Neuroanatomy Lab of China Medical University using microsurgical instrumentation and a surgical microscope (6–40 magnification; OPMI CS-NC; Carl Zeiss).

## Results

### Fiber Dissection Findings

**Figure 2:**
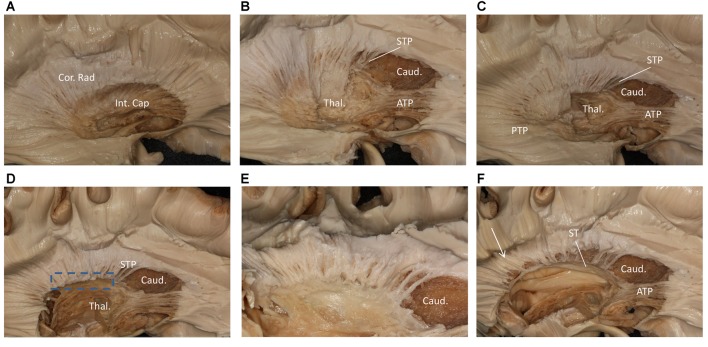
Step by step fiber dissection from lateral to medial of right hemisphere. **(A)** The arcuate fasciculus (AF), superior longitudinal fasciculus (SLF) II and putamen were removed, the internal capsule was exposed. **(B)** After removing the anterior part of internal capsule, the head of caudate nucleus, thalamus, anterior thalamus peduncle and superior thalamus peduncle were exposed. **(C)** The anterior, superior and posterior thalamic peduncle. **(D)** Further removal of the internal capsule and thalamus, we could exposed the most medial part of thalamus peduncle. **(E)** Close-up and bottom to up view of the dotted rectangle in **(D)**, it is clear that lots of fiber bundles reach thalamus. **(F)** After removal of the whole thalamus, some fibers as the white arrow shows coming from the parietal region have a close relationship to stria terminalis (ST). Int. Cap, internal capsule; Cor. Rad, corona radiate; Thal, thalamus; Caud, caudate nucleus; STP, superior thalamus peduncle; ATP, anterior thalamus peduncle; PTP, posterior thalamus peduncle; ST, stria terminalis.

**Figure 3:**
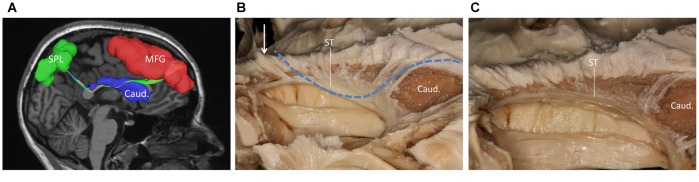
**(A)** Fiber tractography of the SFOF (subject 3, right hemisphere); lateral view of the left SFOF, we found fibers originated from MFG curved around the upper part of the head of caudate nucleus and coursed to SPL. **(B)** The fibers related to ST. **(C)** From** (C)** to **(B)**, we did not find some fiber bundles coursed above the head of caudate nucleus and connected the parietal region as the fiber tractography (dotted line) showed. But we did find some fibers interconnected the parietal lobe with the bottom part of caudate head via stria ternimalis as the white arrow showed. MFG, middle frontal gyrus; SPL, superior parietal lobe; caud, caudate; ST, stria terminalis.

We performed a fiber dissection from the lateral to medial direction. After the arcuate fasciculus (AF), SLF-II and putamen were removed, the internal capsule was exposed (Figure 2A). We then removed the anterior part of the internal capsule, and the head of caudate and the thalamus were then exposed (Figure 2B). The thalamus was medial to the medial and posterior part of the internal capsule, further remove the internal capsule allowing us to visualize the anterior thalamic peduncle (ATP), superior thalamic peduncle (STP) and posterior thalamus peduncle (PTP; Figure 2C). After removal of the internal capsule and thalamus, we were able to expose the most medial part of thalamic peduncles (Figures 2D,E). After removing the whole thalamus, we observed a few fibers coming from the parietal region, which had a close relationship to the stria terminalis (ST; Figure 2F). However, in our fiber dissection, we did not observe any fibers from the frontal lobe that curved around the upper part of the head of caudate and coursed to the parietal and occipital region (as observed by fiber tractography; Figure 3).

**Figure 4:**
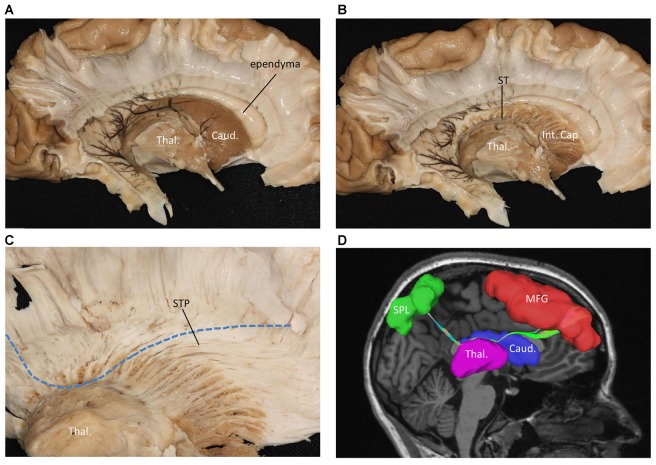
Fiber dissection from medial to lateral of left hemisphere. **(A)** Caudate nucleus and thalamus were exposed. **(B)** After removal of the caudate nucleus, internal capsule and ST were exposed. **(C)** After removal of the ependyma, anterior portion of the subcallosal stratum and the radiation of the CC, we found the superior thalamus peduncle which came from the frontal lobe and reach the superior part of the thalamus. **(D)** Fiber tractography of the SFOF (subject 3, right hemisphere). We did not find an affirmative bundle of fibers interconnect the frontal lobe with parietal region as the dotted line in **(C)** and the SFOF in **(D)** showed. Thal, thalamus; Caud, caudate nucleus; Int. Cap, internal capsule; ST, stria terminalis; STP, superior thalamus peduncle; MFG, middle frontal gyrus; SPL, superior parietal lobe.

We also performed a fiber dissection from the medial to lateral direction. After removal of the cortex, the hippocampus, the CC, and the fornix, the caudate and the thalamus were exposed (Figure 4A). We then removed the caudate, allowing us to see the internal capsule and ST (Figure 4B). Further dissection was performed to identify fibers from the frontal lobe. After we removed the ependyma, the anterior portion of the subcallosal stratum, and the radiation of the CC, we found a few fibers that traversed from the frontal lobe to the superior part of the thalamus, which were likely part of the STP (Figure 4C).

**Figure 5:**
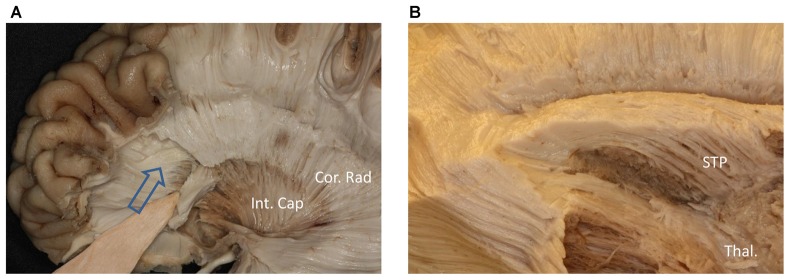
**(A)** Above the head of the caudate and medial to the internal capsule we can see some fibers come from frontal lobe. **(B)** Further dissection shows the fibers come from frontal region and bend inferiorly to the thalamus make up the STP. Cor. Rad, coronal radiation; Int. Cap, internal capsule; Thal, thalamus; STP, superior thalamus peduncle.

During our fiber dissection, we were unable to find a bundle of fibers that coursed around the head of caudate and traveled to the parietal and occipital regions. Above the head of the caudate and medial to the internal capsule, we did observe some fibers coming from the frontal lobe (Figure 5A, arrow). However, further dissection found that the fibers coursed inferior to the thalamus, and were likely part of the STP (Figure 5B).

### Trajectory of the SFOF in the Human Brain

#### Subject-Specific Tractography Findings

**Figure 6:**
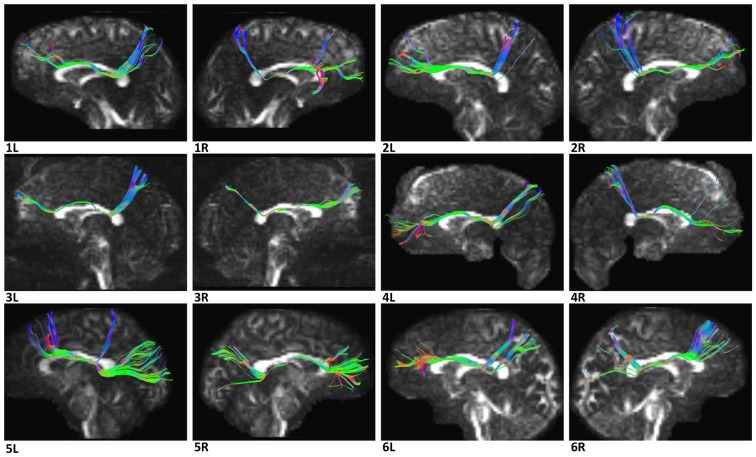
Diffusion spectrum imaging (DSI) tractography of the SFOF in 12 hemispheres of six subjects on sagittal view. Order: subject 1, 2, 3, 4, 5, 6. Left and right SFOF had a similar location, shape and trajectory in all 12 hemispheres. L, left; R, right.

**Figure 7:**
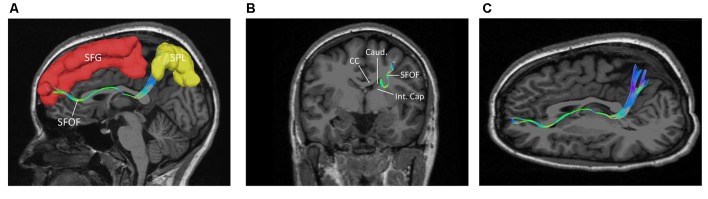
The trajectory and cortical endpoints of the SFOF (subject-specific approach with its T_1_-weighted MPRAGE image; subject 3, left hemisphere). **(A)** Sagittal plane; lateral view of the left SFOF. SFOF originated from the superior frontal gyrus and terminated at the superior parietal lobe. **(B)** In the coronal section, the left SFOF located superolateral to the head of caudate nucleus, under the CC and medial to the internal capsule. **(C)** Combined axial and sagittal view, the left SFOF originated from the SFG and ran posteriorly lateral to the lateral ventricle, and then it ran postero-superiorly and finally terminated in the superior parietal lobule. SFG, superior frontal gyrus; MFG, middle frontal gyrus; SPL, superior parietal lobe; CC, corpus callosum; Caud, caudate nucleus; Int. Cap, internal capsule.

In the fiber tractography study, we found an anteroposterior bundle of fibers passing through the ROIs in 12 hemispheres (Figure 6), which mainly connected the prefrontal cortex with the parieto-occipital region. Based on the cortical parcellation, we performed analysis of the cortical termination of the SFOF (Desikan et al., 2006). This distinct group of fibers mainly originated from the superior frontal gyrus (SFG) and middle frontal gyrus (MFG), and formed a compact fascicle at the level of the anterior horn of the lateral ventricle. The fibers ran posteriorly under the CC, medial to the internal capsule and lateral to the lateral ventricle, and then ran postero-superiorly, as they terminated mainly at the superior parietal lobule and precuneus (Figure 7).

#### Template Tractography Findings

**Figure 8:**
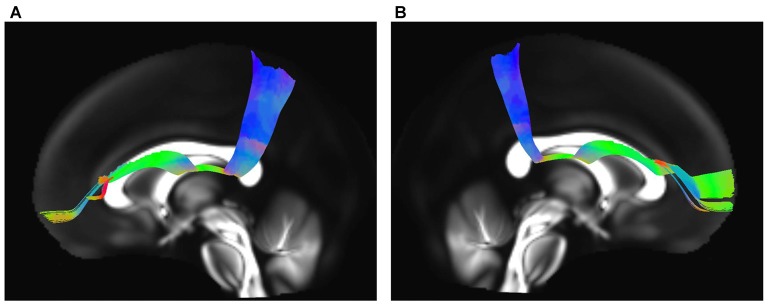
*In vivo* fiber tractography of the SFOF on Human Connectome Project (HCP-842) template. **(A,B)** SFOF in HCP-842 template and the HCP-842 template showed similar results to the subject-specific findings. L, left; R, right.

The DSI template (HCP-842) showed similar results to the subject-specific findings above (Figure 8).

### Spatial Relationship of the SFOF with Adjacent Association Tracts

**Figure 9:**
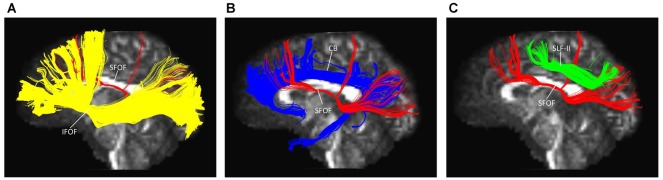
Spatial relationship of the SFOF with the adjacent association tracts. **(A)** Sagittal view showed the SFOF located above the inferior fronto-occipital fasciculus (IFOF). **(B)** Sagittal view showed the cingulum bundle (CB) was above and medial to the SFOF. **(C)** Sagittal view showed the SLF-II was above and lateral to the SFOF. SFOF, superior fronto-occipital fasciculus; IFOF, inferior fronto-occipital fasciculus; CB, cingulum bundle; SLF, superior longitudinal fasciculus.

The IFOF, SLF-II and CB were reconstructed to show the spatial relationship of the SFOF with adjacent association tracts (Figure 9). The IFOF passed from the prefrontal region to the occipital region, and the external/extreme capsule level fibers of the IFOF narrowed into a compact fascicle, forming the stem of the IFOF (Wu et al., 2016a). We also simultaneously reconstructed the IFOF and SFOF in one hemisphere, and found that the stem of the SFOF was located above the stem of the IFOF (Figure 9A).

The CB is a critical white matter fiber tract in the brain, which forms connections between the frontal lobe, parietal lobe, and the temporal lobe (Wu et al., 2016b). We simultaneously reconstructed the CB and SFOF in one hemisphere, and found that the CB was a sickle-shaped association bundle that nearly encircled the CC and extended to the temporal lobe, and that the CB was located above and medial to the SFOF (Figure 9B).

The SLF-II is a component of the SLF that runs on the lateral aspect of the hemisphere and connects the parietal lobe with the caudal MFG and the dorsal precentral gyrus. We simultaneously reconstructed the SLF-II and SFOF in one hemisphere, and found that the SLF-II was located superolateral to the SFOF (Figure 9C).

## Discussion

Since Dejerine (1895) reported that SFOF exits in the health human brain, SFOF has never been identified in recent fiber dissection studies (Türe et al., 1997; Meola et al., 2015). We did not find a bundle of fibers corresponding to the SFOF in our fiber dissection study either. In the ROI, we found fibers coming from the frontal lobe that coursed above the head of caudate and media to the internal capsule. However, further dissection demonstrated that these fibers belonged to the STP. During our lateral to medial fiber dissection, after removal of the whole thalamus we found some fibers coming from the parietal region that were not the PTP, but which had a close relationship to the ST. However, because of the crossing fibers and complicated structure in this region, we could not identify a distinct bundle of fibers coming from the frontal lobe and connecting with these fibers.

In contrast to our fiber dissection findings, we reconstructed the SFOF in six subjects and in a template of 842 subjects based on the DSI method. We found that the shape and trajectory pattern of the SFOF were similar to previous DTI studies (Catani et al., 2002; Makris et al., 2007). However, recently Meola et al. (2015) reported that prior SFOF reconstructions based on DTI method were generated by false continuations between the STP and ST, and between ST and the PTP. So, can we trust our findings based on DSI tractography? We think we can’t fully trust the findings of our DSI tractography study, on the one hand, we did not identify the SFOF in our fiber dissection study; on the other hand, any diffusion tensor tractography may have a false connection due to the biological signals noise, partial volume effects, magnetic resonance resolution and tracking algorithm (Chen and Song, 2008; Mohammadi et al., 2013). However, fiber dissection technique also has limitations for resolving crossing fibers and following long fibers (Wang et al., 2016). Besides, the individual skills and anatomical knowledge of the investigators may affect the fiber dissection results. We think the results of our DSI tractography study also have some significance. Schmahmann et al. (2007) used DSI and isotope tract-tracing (gold standard) to trace long association fibers in the monkey brain, are found that the two methods showed a remarkable concordance. In our tractography study, to filter out the fibers that belonged to STP and PTP, we set the middle ROI above the thalamus on the sagittal plane, and in the same position as the rostral and caudal ROIs on the coronal plane (Figure 1C). Using three ROIs, we reconstructed the SFOF that coursed above the dorsal thalamus (Figure 4D), which was distinguishable from the STP-ST-PTP. If the DSI tractographic SFOF really exist, based on cortical parcellation (Desikan et al., 2006), we found that the SFOF would originate from the rostral and medial parts of the superior and MFG (Brodmann area [BA] 8, 9 and 10), the fibers formed a compact fascicle at the level of the anterior horn of the lateral ventricle coursing above the head of caudate, medial to the corona radiate and under the CC, and terminated at the superior parietal lobe (SPL) and precuneus (BA area 7) via the lower part of the caudate. These DSI data provide a more precise trajectory and connectivity pattern than previous DTI studies (Catani et al., 2002; Makris et al., 2007). Because of the limitations of DTI tractography, Makris et al. (2007) could not confirm the origins and terminations of the SFOF in humans, rather they extrapolated these from experimental data in monkeys. By contrast, our findings are based on advanced HARDI, which solves the crossing fiber problem and provides detailed evidence of the cortical site of origin or termination of the SFOF without the need for approximation (Fernandez-Miranda, 2013; Wu et al., 2016b).

Literally speaking, SFOF is a bundle of fibers connecting frontal and occipital lobes, however, our tractography study and some previous DTI studies (Catani et al., 2002; Makris et al., 2007) show that the SFOF mainly connects frontal lobe with parietal region. If the SFOF really exists in humans, we think that the differences between our result and previous studies of classical neuroanatomy and monkey brain may due to the limitation of gross dissection and brain evolution of human beings.

Despite reconstructing the SFOF based on advanced HARDI in the present tractography study, we cannot conclude that SFOF exists in the human brain as we did not find it by fiber dissection, and there is also potential for false connections in tractography data. The combination of *ex vivo* DSI scans with fiber dissection in the same human brain, or of *in vivo* DSI scan with fiber dissection in the same non-human primate brain, the comparison will be more compelling. In addition, new method like polarized light imaging (PLI), which can identify nerve fibers and their spatial orientation in microtome sections of postmortem brains based on the birefringent properties of the myelin sheaths (Dammers et al., 2012; Mollink et al., 2017), can further verify our DSI tractography study.

There were several limitations in our study. First, a subject-specific approach was completed in only six subjects. More subjects are required to manifest whether the SFOF can be reconstructed in each subject. Second, although fiber dissection can provide key macroscopic information on fiber tract anatomy, it has limited value for the study of white matter trajectory in areas of crossing fibers. Finally, the potential functional role of the SFOF in the present study is only based on correlation analysis, and remains to be proven.

## Conclusion

Using HARDI tractography, we identified the SFOF in six human subjects, and described the trajectory and cortical endpoint of the SFOF. Based on its shape, trajectory, and anatomical relationship with related structures, we consider that this fiber bundle observed by tractography may be the SFOF, although it terminates mainly at the parietal region, rather than occipital lobe. Nevertheless, we were unable to identify a fiber bundle corresponding to the SFOF in our fiber dissection study, which is likely because of limitations in the fiber dissection technique. Although we did not provide definite evidence of the SFOF in the human brain, these findings may be useful for future studies in this field.

## Author Contributions

YiW and YB: conceived and designed the experiments, wrote the article and revised the manuscript. YB, YiW and YoW: performed the experiments. YB and WW: data interpretation and picture preparation. YiW and YoW: collected the biopsy samples. YiW and WW: contributed reagents/materials/analyses tools.

## Conflict of Interest Statement

The authors declare that the research was conducted in the absence of any commercial or financial relationships that could be construed as a potential conflict of interest.

## Footnotes

^1^http://surfer.nmr.mgh.harvard.edu
